# Medicine as a Career

**Published:** 1914-09-05

**Authors:** 


### The Hospital

The Workers' Newspaper of Administrative Medicine and Institutional Life, Administration, National Insurance and Health.

No. 1472, Vol. LVI. SATURDAY, SEPTEMBER 5, 1914. PRICE ONE PENNY.

### EDUCATIONAL NUMBER.

### MEDICINE AS A CAREER.

The prospects offered by a medical career have to be considered in a certain number of households even amidst the sounds and noise of war. The civic, social, and professional activities of the nation must be carried on. Recruits are needed for all of these as well as for the Army. Adventurous youth pushes ever to the margin of decision. Which path shall be selected, and which goal preferred, are questions that recur without cessation as the generations continue and renew themselves. And among the paths to be considered is that offered by the profession of medicine.

A general remark often made, and yet well to be repeated, is that medicine is no career for unwilling recruits. It presents, without doubt, intellectual and moral interests which appeal to certain minds with an almost overwhelming fascination, and to such there is no denial. They can and will no other. But with equal certainty there are others to whom the medical life, so far from being attractive, is positively repellent, and to these we have no hesitation in saying that they will be well advised to hold to their individual inclinations, and to resist all circumstances and all advice which would urge them into the medical ranks. Between these two extremes there are intermediate groups with no very decided choice either one way or the other, and here family and other influences are likely to exercise much sway. Even for these the remark already made remains true. If in doubt they had better remain outside. Medicine at its best, and we desire to contemplate nothing less, calls for high service and whole-hearted devotion, and those who have not such to give can scarcely look either for satisfaction or for success.

In insisting on what we have just written we are far from wishing to deter anyone from following an inclination to select medicine as his life's work. The responsibilities are considerable, and anything like great financial success is in the highest degree improbable. On the other hand, there are for those who are strong and resolved abundant compensations. The work is rich in intellectual discipline, and is touched at all points with the sense and emotion of human relationship. It is highly conducive to personal culture and to the growth of such qualities as tact, patience, resolution, courage, and good will. And in a very special degree it gives to life what is one of the great boons—a sustained and never-failing stimulus to high endeavour. Here, perhaps more than in any other career, may be heard without ceasing the apostolic injunction to covet earnestly the best gifts. Therefore let those who feel a prompting to this choice be not afraid. Rather we would urge them to a cheerful courage and to a conviction that great things are possible to those of resolute heart and of clear and undismayed vision.

It may be useful briefly to consider what influence recent legislative and other changes have established on the position and outlook of the medical profession. Speaking generally, these changes have tended to enlarge and emphasise the public relations of medicine. The large questions of preventive medicine, and the national importance of the public health, have become increasingly prominent, and in this direction expert professional opinion has touched closely both legislation and administration. Hence the medical officer of health has become both more independent and more important, and, as a result, every year a certain number of practitioners enter the public service almost immediately after graduation with the expectation of rising to the more responsible and better paid positions. There is here quite a reasonable prospect of a fair income and a respected position for any practitioner who adds to the necessary technical equipment the gifts of tact and administrative ability. In this direction new and special problems are continually arising, and special ability and industry are sure to have their opportunity. To illustrate this statement we may quote one or two of the modern developments in connection with tropical medicine. Here especially has been fruitful the search for the causes of disease, for the discovery of causes is promptly followed by attempts at prevention, and is a necessary preliminary to such attempts. Take as a simple illustration the case of malarial fever. So long as the cause was unknown the activities of medicine were confined to the cure of individual men and women. But the discovery once made that the cause of the disease is a living organism which is introduced into the blood by the bite of a particular species of mosquito, and a much wider horizon presents itself. The question is now of the means by which such infection can be prevented. Instead of the successful treatment of an individual, the ambition of medicine enlarges itself into an endeavour to protect a whole community, and this, in practice, has meant the establishment of healthy conditions in parts of the globe where previously the inhabitants in very large proportions were the victims of a serious and often fatal scourge. It is a somewhat trite observation, but it is none the less true, that no engineering skill could have constructed the Panama Canal had not medical science first made it possible for labourers to live on the fever-smitten isthmus. To come nearer home, a similar problem is now being faced in the case of tuberculosis. The cause of the disease is known. It remains to find practical administrative methods by which prevention can be effected. Here, then, are examples of the wide reach which medicine in its preventive aspects has attained, and no one need fear that the field of opportunity will be readily exhausted.

Another illustration of the growing public relations of medical science is the demand for the official examination of school children, with the almost necessary corollary of the provision of adequate treatment at the public expense. Such a position is already fully attained in connection with tuberculosis, and its extension to other diseases is practically inevitable. Further, out of such inspection there must needs arise the question of prevention, with its relation to such influences as housing, food, and clothing. Not only the public conscience, but equally the national welfare, cannot long tolerate the existence in our midst of widespread preventable disease. There is thus a growing demand for the application of medical knowledge to problems which formerly were regarded as the concern of the individual, or, at the best, the concern of the charitable public. A healthy and vigorous community is seen to be a national asset of the first importance, and an appropriate subject therefore for legislative interference and for public money. In the case of infectious diseases every citizen now accepts this proposition as a commonplace, and an extension of the principle to various other conditions is morally certain. In short, to a greater and greater extent medicine is recognised as concerned with national welfare, and with this there is necessarily a wider sphere of opportunity for the medical practitioner.

As a third illustration of the increasing application of medicine to larger problems than the cure of the individual may be mentioned the wide extension of the principle of insurance. Such legislative enactments as the Employers' Liability Act and the Workmen's Compensation Act are constantly raising questions which only medical knowledge can solve. And as a result quite a number of practitioners are to-day occupied mainly, or even entirely, in dealing with such issues, and are justly earning the fees which expert skill and knowledge command. We are not now concerned with controversy, but it will not be questioned that one of the aims of the National Insurance Act was to secure, and partly at the public expense, improved medical treatment for the poorer classes of the community, and this, not merely for the benefit of the individual sufferer, but in the hope of advantage to the community in general by the establishment, so far as possible, of methods tending to limit and to prevent disease. This, again, means the recognition of the wide range and play of medical knowledge and of the necessity of the application of such knowledge on a large scale in the interests of the public welfare.

The bearing of all that we have so far written on the fortunes of those who are now about to enter on a career of medical study is briefly this, that medicine to-day is offering larger occasions for public service and that, to a greater extent than has hitherto been the case, the newly qualified practitioner may count upon the certainty of an income which will at least give him a secure livelihood and may well be the earnest of still better things.

To insist upon the newer opportunities which modern medicine offers must not, however, be taken to mean that individual practice is failing and is likely to disappear. However effective may be our preventive measures, there will still remain sickness and disease, not to speak of the accidents and emergencies of daily life. Hence the private practitioner will continue to flourish, and, in our view, greatly to the advantage of the community. Like other institutions, he will change with changing times, and thus, in accordance with his training, his work, we anticipate, will take on a more scientific quality. Most of us of the passing generation had but little training in laboratory methods or in the use of instruments of precision as aids to diagnosis and treatment. These are now prominent parts of the student's education, and the more thoroughly he learns them the more efficient, self-reliant, and trustworthy a practitioner will he become. Here are abundant opportunities for worthy service, and to such ambitions we bid our young *confrères* a cordial welcome. They are heirs to great traditions, and we doubt not they will prove themselves a loyal succession.

